# Controlled Delivery of Celecoxib—β-Cyclodextrin Complexes from the Nanostructured Titanium Dioxide Layers

**DOI:** 10.3390/pharmaceutics15071861

**Published:** 2023-07-01

**Authors:** Magdalena Jarosz, Jakub Latosiński, Paweł Gumułka, Monika Dąbrowska, Mariusz Kępczyński, Grzegorz Dariusz Sulka, Małgorzata Starek

**Affiliations:** 1 Department of Physical Chemistry & Electrochemistry, Faculty of Chemistry, Jagiellonian University, Gronostajowa 2, 30-387 Krakow, Polandsulka@chemia.uj.edu.pl (G.D.S.); 2 Department of Inorganic and Analytical Chemistry, Faculty of Pharmacy, Jagiellonian University Medical College, 30-688 Krakow, Poland; pawel.gumulka@student.uj.edu.pl (P.G.);; 3 Doctoral School of Medical and Health Sciences, Jagiellonian University Medical College, Łazarza St., 31-530 Krakow, Poland

**Keywords:** inclusion complexes, celecoxib, β-cyclodextrin, nanostructured titanium dioxide, drug delivery

## Abstract

Considering the potential of nanostructured titanium dioxide layers as drug delivery systems, it is advisable to indicate the possibility of creating a functional drug delivery system based on anodic TiO_2_ for celecoxib as an alternative anti-inflammatory drug and its inclusion complex with β-cyclodextrin. First, the optimal composition of celecoxib—β-cyclodextrin complexes was synthesized and determined. The effectiveness of the complexation was quantified using isothermal titration calorimetry (ITC), differential scanning calorimetry (DSC), infrared spectroscopy (FT-IR) nuclear magnetic resonance (^1^H NMR), and scanning electron microscopy (SEM). Then, nanostructured titanium dioxide layers (TiO_2_) were synthesized using the electrochemical oxidation technique. The TiO_2_ layers with pore diameters of 60 nm and layer thickness of 1.60 µm were used as drug delivery systems. The samples were modified with pure celecoxib and the β-cyclodextrin-celecoxib complex. The release profiles shown effective drug release from such layers during 24 h. After the initial burst release, the drug was continuously released from the pores. The presented results confirm that the use of nanostructured TiO_2_ as a drug delivery system can be effectively used in more complicated systems composed of β-cyclodextrin—celecoxib complexes, making such drugs available for pain treatment, e.g., for orthopedic surgeries.

## 1. Introduction

Celecoxib (CEL) has been recognized for many years as an important drug in treating postoperative pain. Numerous scientific studies support its effectiveness; therefore, its use is recommended by many pain-management societies [1]. The main benefit of CEL is a reduction in the use of opioid drugs. Schroer et al. reported research involving 107 patients, where the use of CEL for 6 weeks after knee arthroplasty was associated with less perioperative opioid use and better results on the Visual Analog Scale (VAS) [2,3]. Several clinical trials led to similar conclusions, for example, a clinical trial called “PIPFORCE”, which included patients undergoing total knee replacement surgery [4,5]. Another benefit of the use of coxibs in the confirmed reduction in the incidence of post-operative cognitive dysfunction and heterotropic ossification after total arthoplasty [6,7].

Currently, CEL is a drug available on the market only as oral tablets. However, there are attempts to modify the route of administration and the rate and mode of its release. Tellegen et al. attempted to deliver CEL locally to the intervertebral discs to relieve inflammation and prevent back pain [8]. Additionally, it was necessary to modify the release rate. Closing the drug in polyesteramide microspheres resulted in a permanent inhibition of inflammation, as evidenced by the reduced production of prostaglandins and anti-catabolic activity in the nucleus pulposus. In vivo studies in dogs showed that the controlled release of CEL from the microspheres in the intervertebral disc area prevented the progression of its degeneration. The Willems’ team also explored the possibility of the topical application of CEL to the intervertebral discs [9]. In this case, the vehicle responsible for the sustained release of CEL was a thermoreversible hydrogel. Another example of topical CEL administration was a study aiming to determine the possibility of drug release from intraocular lenses [10]. CEL is effective in the treatment of secondary lens opacities following cataract surgery. Intraocular lenses incubated in CEL solution for 24 h resulted in sustained in vitro drug release at levels sufficient to inhibit the increase in opacity. Recent studies have shown that CEL may also have antitumor activity, so research was undertaken to create the possibility of administering the drug intravenously. Due to the fact that CEL is poorly soluble in water, it must be combined with a carrier facilitating its solubility. A research team of Xv et al. developed a method of saturating casein nanoparticles stabilized with phosphatidylcholine with CEL [11]. Studies on rats have also confirmed the clinical effectiveness of the obtained formulation. Based on the information presented above, searching for new administration forms of this drug and modifying its release rate is necessary.

Cyclodextrins (CDs) are cyclic oligosaccharides linked by α-1,4-glycosidic bonds [12]. Due to the hydrophilic outer surface, they are well soluble in water, while the inner space is hydrophobic and may contain another hydrophobic substance, forming an inclusion complex. They have the capacity to construct inclusion complexes with various guest molecules on account of their exceptional truncated cone structure [13]. CDs are widely used in pharmacy, e.g., as drug carriers to modify the bioavailability of biologically active molecules [14], or to improve their physicochemical properties, including increasing stability or solubility [15]. Due to these properties, it seems advantageous to use a complex of CD with CEL. Nagarsenker et al. created solid dispersions of CEL with hydroxypropyl-β-cyclodextrin (HP-β-CD) by various methods: as a physical mixture, by co-milling, kneading, or co-evaporation [16]. The kneaded form resulted in the highest dissolution rate and better release profile compared to pure CEL. In vivo studies in rats revealed that the kneaded dispersion provided a faster response and was more effective in inhibiting rat-paw edema compared to CEL alone. Another team studied the solid complexes of CEL and β-CD. Their formation by lyophilization, evaporation and kneading were compared, and the solubility tests were performed. A significantly higher rate of dissolution was demonstrated for the complexes compared to the pure drug and its physical mixture [17]. Jansook et al. created CEL eye drops. In order to deliver this practically water-insoluble drug to the ocular space, a triple complex (CEL, β-CD, polymer) was made by sonication [18]. The results indicated that the obtained mixture resulted in higher drug permeability through a semi-permeable membrane simulating the vitreous and sclera tissues. Such complexes have also been shown not to be cytotoxic to human retinal cells. The possibility of the transdermal administration of CEL was also assessed. The in vivo activity of multi-vesicle liposomes containing the β-CD, CEL–β-CD inclusion complex was observed by evaluating the anti-inflammatory effect of rat-paw volumetric edema. The prolonged-action preparation obtained in this way showed an activity of up to 120 h [19]. In another study, the authors compared chitosan microparticles, saturated with CEL and CEL in a complex with a β-CD derivative, for intravesical administration [20]. In an in vitro release study, an immediate full-dose release was demonstrated for the complex and sustained release of CEL alone. Research proved that by using different saturations of nanoparticles, it is possible to obtain a modified drug release rate. One of the main advantages of using complexes with CD is the increase in the bioavailability of a drug. Rescifina et al. investigated the effect of complexing CEL with sulfobutyl ether-β-CD to make an inhalable dry powder formulation of gemcitabine for lung cancer therapy [21]. CEL alone displayed low cytotoxicity to A549 cell lines, and the complexation increased lung cancer cell death. The presented studies confirm the advantages of inclusion complexes that improve the physicochemical properties of CEL, which positively affect its local action. Without this modification, in many cases it would be impossible to obtain a local drug effect in many cases.

As mentioned above, CEL may be used in therapies after orthopedic surgeries. In the case of bone or dental implantations, the standard treatment requires oral or intravenous administration of the drugs, which requires high dosages and repetitions. This solution is not ideal for the patient and is ineffective. Therefore, the local delivery of the medicaments is being searched. Among different implant materials, titanium and its alloys are the most commonly used materials in dentistry and orthopedics [22,23]. Such highly biocompatible materials have very good mechanical properties and relatively good corrosion resistance [24]. Due to the passivation process, an oxide layer is formed, facilitating the connection between cells and the implant surface [25]. The downside of such biomaterials is that the osseointegration process takes time, which may lead to inflammation or even implant rejection.

One proposed solution for improving titanium-based implants is forming a nanostructured titanium dioxide layer before implantation [25,26,27]. Among various available methods for its synthesis, the electrochemical oxidation (also known as anodization) of titanium support is one of the most versatile techniques. The main advantage of the anodization is that by changing the process conditions, e.g., applied potential, time, and composition of the electrolyte; one can manufacture oxide layers characterized by designed morphology, i.e., with a specified pore diameter (D_p_), an oxide layer thickness, porosity, etc. [28,29]. For example, when fluoride ions are added to the electrolyte, nanotubular or nanoporous oxide layers are obtained. Furthermore, when we change the applied potential between 30 and 70 V, pore diameters change in the range of around 50 to 80 nm, while the layer thickness varies from approx. 1.3 to 7.3 µm [29]. Due to the increased possibilities in modifying titanium and titanium-alloy surfaces with oxide layers with versatile nanotopographies, such materials have been successfully tested for their applicability as biomaterials [30,31]. It was shown that Ti-based implants modified with a nanostructured TiO_2_ layer were characterized by increased biocompatibility and superior osteointegration compared to non-modified supports. What is more, the cell response to the surface significantly depends on the nanotopography of the layers, especially on the pore diameters. For example, Park et al. show a significant correlation between pore diameter and cell fate and that the optimal geometry of the nanostructured titanium dioxide layers for the cell’s growth and differentiation should be between 30 and 50 nm [32]. On the other hand, Brammer et al. presented results where the optimal pore diameter was 100 nm; mesenchymal stem cells were significantly more elongated and presented with a higher expression of alkaline phosphatase activity, which proved that nanotubes with higher diameters had increased bone-forming abilities [33]. That only suggests that nanotopography of the titanium dioxide layers is extremely important for osteointegration and bone formation around the implantation site, whereas the nature of this impact is still not explicit.

A cells’ fate depends not on the size and length of the nanostructures alone. Their presence allows one to fill them with drugs and makes them an excellent on-site delivery system. Many studies show that nanostructured titanium oxide layers may be used as efficient drug delivery systems (DDSs) with controlled and prolonged delivery characteristics [34,35,36,37,38,39]. Similarly to studies on the interaction between cells and TiO_2_ surface, also in the case of drug delivery systems, the topography of the nanostructured layers will play an important role. The pore diameter and depth of the nanotubes/nanopores will impact the amount of the drug loaded inside the pores and the release kinetics [40,41,42]. Also, other parameters like the concentration of the drug, surface wettability, and the nature of the drug, i.e., its hydrophilicity/hydrophobicity, as well as the molecule’s size, may have an impact on both the loading and releasing of the molecules from the nanostructured delivery system [43,44]. Moreover, the surface of titanium dioxide layers may be further modified in order to inhibit the release of the drugs. Thus, TiO_2_ layers may be modified with different polymers [45,46] or other compounds like hydroxyapatite or silane derivatives [47,48] that will enable the release of the drugs but at a significantly slower rate.

Considering the potential of nanostructured titanium dioxide layers as DDS and CEL as an alternative anti-inflammatory therapeutic, this study aimed to show the possibility of creating a functional DDS based on anodic TiO_2_ for the CEL and its inclusion complex with β-CD. To the best of our knowledge, this is the first time such an approach for CEL delivery has been undertaken. For the presented preliminary studies, titanium dioxide layers with pore diameters of around 50 nm were used as reservoirs for the delivery of CEL and CEL–β-CD complexes. The formation of inclusion complexes was optimized, and the optimal formulation was used for further studies. The loading procedure of both molecules was optimized, and the delivery of CEL was conducted for 24 h in phosphate-buffered saline. Our results show that CEL can be delivered to the site using the nanostructured oxide carrier in the form of an inclusion complex which has enhanced solubility. These studies are an important step into adapting the method of drug administration to the systemic circulation (released directly at the postoperative site) to enhance the analgesic effect or relieve acute pain.

## 2. Materials and Methods

### 2.1. Isothermal Titration Calorimetry (ITC) Measurements

The measurements were performed using a Microcal PEAQ-ITC calorimeter (Malvern Instruments Limited, Worcestershire, UK) equipped with two 200 μL cells. A total of 19 injections of 2 μL each (the first injection of 0.4 μL) of the β-CD solution (15 mM) in the water-methanol mixture were made to the CEL solution (0.992 mM) in the water-methanol mixture, injection duration = 4 s, at 25.2 °C. The interval between injections was 150 s, and the stirring speed was 500 rpm. The heats were corrected for the dilution effects determined in separate experiments. Data were analyzed using MicroCal PEAQ-ITC analysis software. The “one set of sites” mathematical model was applied to analyze the thermodynamic parameters.

### 2.2. Differential Scanning Calorimetry (DSC)

To perform the thermal analysis a Q200 calorimeter (TA Instruments, New Castle, DE, USA) was used. Test samples weighing 2 mg were closed in aluminum pans (TzeroPan), and heated from 30 to 180 °C in a nitrogen atmosphere. The heating speed was 5 °C/min. An empty pan was used as a reference and physical mixtures were used as control samples.

### 2.3. Fourier-Transform Infrared Spectroscopy (FT-IR)

The interactions between CEL and β-CD were characterized by FT-IR spectroscopy using a Thermo Nicolet iS10 FTIR spectrometer (Thermo Scientific, Waltham, MA, USA) with an ATR accessory (SMART iTX) in the interval of 600–4000 cm^−1^, at an optical resolution of 4 cm^−1^. All the samples (CEL, β-CD, CEL–β-CD) were analyzed after vacuum drying. The obtained spectra were baseline corrected and normalized using Omnic v9.0 software (Thermo Scientific).

### 2.4. Proton Nuclear Magnetic Resonance (^1^H NMR)

Proton NMR spectra were measured with FT-NMR 500 MHz JEOL instrument (JNM-ECZR500RS1 v. ECZR). ^1^H NMR spectra were recorded at temperature 25 °C, at 8 scans, and ROESY (Rotating Frame Overhauser Enhancement Spectroscopy) at 32 scans and 256 repetitions, mixing time 25 ms. Deuterated dimethyl sulfoxide (DMSO-d_6_) was used for experiments as a solvent.

### 2.5. Scanning Electron Microscopy (SEM) and Energy Dispersive X-ray Spectroscopy (EDS)

The microscopic changes of the tested samples were evaluated using SEM and EDS techniques. CEL, β-CD, and complex powders were mounted on the carbon adhesive tape and examined under the microscope. For each sample, microphotographs were taken along with the EDS spectra showing the element distribution.

### 2.6. Synthesis of Nanostructured TiO_2_ on Ti Support

Nanostructured TiO_2_ on a titanium support (TiO_2_@Ti) was prepared using the electrochemical oxidation (i.e., anodization) method with the procedure commonly used in the Electrochemistry Group, JU [29]. Briefly, Ti foil was cut into coupons with dimensions of 2 × 1 cm and pressed with the manual press, so the samples were flat. Then, Ti samples were polished according to the procedure established by Jarosz et al. [49]. Next, polished Ti coupons were used as anodes in the three-step anodization process carried out in the ethylene glycol-based electrolyte. The process conditions are given in Table 1.

After the process, samples were washed in distilled water, dried in the air, and weighted.

The topography of the TiO_2_ samples was evaluated using scanning electron microscopy (FE-SEM Hitachi S-4700 with Noran system) and evaluated with WSxM 5.0 Develop 9.3 software [50].

The water-contact angle on the tested samples was measured using an OCA25 goniometer (DataPhysics) with an automatic dosing system. At least three separate water droplets (droplet volume was 2 µm) were put on the surface of anodic TiO_2,_ and the contact angle was measured using the provided software. Moreover, the surface energy was calculated using the water and diiodomethane contact-angle measurements. The Owens, Wendt, Rabel & Kaelble (OWRK) method was used to calculate the surface energy.

### 2.7. Modification of TiO_2_@Ti with Tested Molecules

TiO_2_@Ti samples were used as drug delivery systems. For that reason, samples were modified with CEL or inclusion complex (CEL–β-CD) following the loading procedure implemented for other drugs [42,51]. Firstly, the drug solutions with 1 mg/mL concentration were prepared in methanol and the mixture of methanol and water (1:1 *v*/*v*) for CEL and the complex, respectively. Afterward, portions of 200 µL were pipetted on the surface of TiO_2_ and left for the solvent to evaporate. The procedure was repeated 5 times to insert the total volume of 1 mL of drug solution into the nanostructures. It is worth noting that the samples were weighted after each cycle to monitor the changes in the mass of the loaded molecules.

### 2.8. Drug Release and Determination

Modified TiO_2_ supports were put in the 15 mL Falcon tubes filled with 10 mL phosphate-buffered saline (PBS) with a pH of 7.4. Drug release was performed at 37 °C for 24 h, where the whole volume of the medium was gathered at the pre-determined time points and replaced with the fresh portion of the solution. For each drug, the measurements were performed in triplicates, and the presented results are the mean values of those measurements.

The determination of the drug concentration was performed using the thin layer-chromatography technique (TLC). The applied method of coxibs determination was fully validated according to ICH requirements. The developed conditions allow for obtaining repeatable, accurate, and reliable determinations results, enabling the qualitative and quantitative analysis of ingredients [52]. Separation was performed on 20 × 10 cm aluminum sheets precoated with silica gel 60F_254_ plates. Samples were applied to the plates as bands (5 mm wide and 10 mm apart) by a Linomat V sample applicator (CAMAG, Switzerland) equipped with a 100 μL syringe (Hamilton, Switzerland) with an application rate of 200 nL/s. The first application was 10 mm from the bottom edge and 10 mm from the left edge of the plate. The volume of the applied solution was 50 µL. Plates were taken into a chromatographic chamber (18 × 16 × 8 cm; Sigma-Aldrich, St. Louis, MO, USA) previously saturated with mobile phase vapor for 20 min at room temperature. Correct separation and well-developed peaks were obtained with a mobile phase containing chloroform: acetone: toluene (12:5:2, *v*/*v*/*v*). The development distance was 10 cm over 20 min. After development, plates were dried at room temperature for about 20 min. Densitometric detection was performed using a TLC Scanner 3 with winCats 4 software (CAMAG, Muttenz, Switzerland). The radiation source was the deuterium lamp emitting a continuous UV spectrum between 200 to 400 nm. The scanning speed was 20 mm/s, and the slit dimension was 4.00 × 0.45 mm. Based on the absorption spectra, the analytical wavelength of 254 nm was selected for measurements.

## 3. Results and Discussion

First of all, we decided to form a complex between CEL and β-CD. The process of optimizing the synthesis conditions led to the selection of the kneading method as the most effective. The success of the CEL–β-CD complex synthesis was confirmed by thermal analysis (DSC) and spectroscopic methods (FT-IR, ^1^H NMR). In the next stage of research, nanostructured titanium dioxide (TiO_2_) layers were synthesized and used as a drug delivery system. TiO_2_ layers were modified with pure CEL and the β-CD–CEL complex, and then drug release experiments were conducted.

### 3.1. Synthesis and Characterization of Celecoxib–β-Cyclodextrin Complexes

Isotermal titration calorimetry (ITC) measurements were used to quantify the association of CEL with β-CD. The ITC method measures the heat released when an aliquot of the β-CD solution is injected into the CEL solution. The ITC data are integrated over time to obtain the incremental heat versus the β-CD/CEL molar ratio. After correcting for the heat of dilution, the incremental heat for each injection is fitted to an appropriate equilibrium binding model to determine the enthalpy of association (Δ*H*), association constant (*K*_A_), and the stoichiometry of association (*n*) [53,54,55]. Since measurements are made at a constant temperature, the change in the Gibbs free energy (Δ*G*) and entropy (Δ*S*) can be calculated using the standard Equations (1) and (2):

(1) $$∆G=-RTlnK_{A}$$

(2) $$∆S=\frac{∆H-∆G}{T}$$

**Figure 1:**
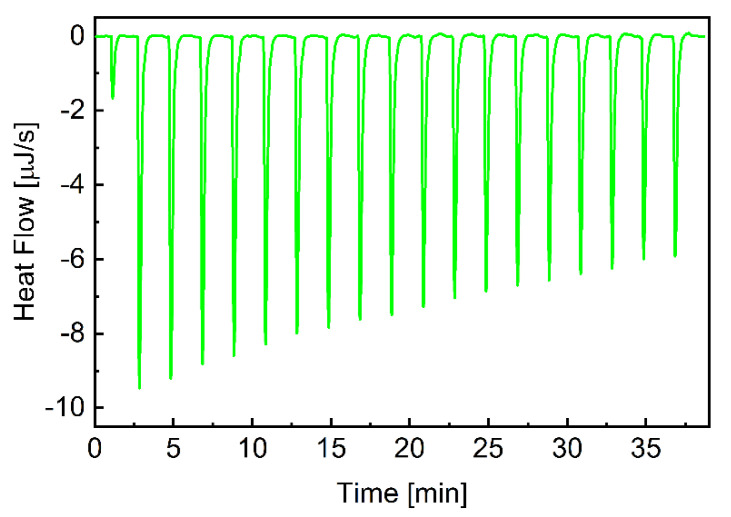
ITC plots for β-CD association with CEL. The heat flow for consecutive injections of the β-CD solution into CEL. Experiments were performed at 25.2 °C.

**Figure 2:**
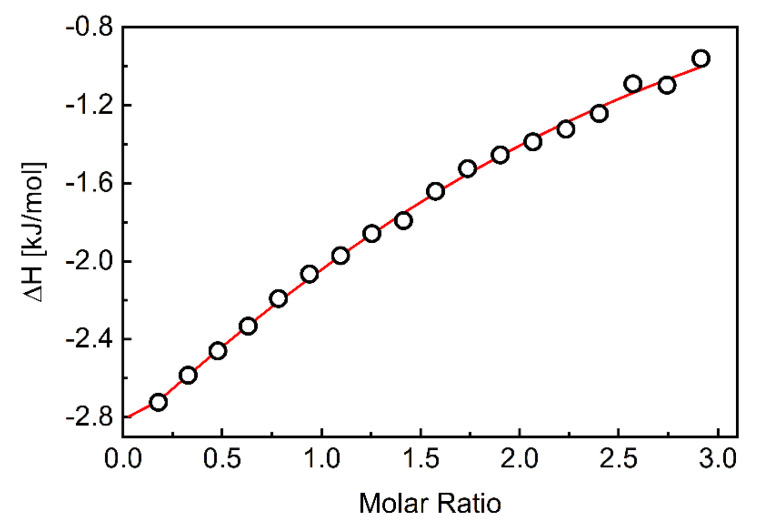
The integrated heats of each injection (corrected for dilution effects) as a function of the β-CD/CEL molar ratio. The red line corresponds to the best fit using a MicroCal PEAQ-ITC analysis software based on the one-site binding model.

Figure 1 depicts a typical thermogram of the β-CD-to-CEL titrations, which reveals that the association of the drug to β-CD is an exothermic process. Each injection of the β-CD solution into the drug solution led to a fast exothermic heat flow, but the observed thermal effect decreased with successive injections. This indicates an equilibrium between CEL molecules interacting with the CD cavity and those remaining in the water. Titration curves as a function of β-CD/CEL molar ratio were obtained by integrating the heat pulse of each injection concerning time and dividing it by the number of moles of the injected agent (Figure 2). The isotherms were fitted using a single set of the independent binding sites (SSIS) model [51], yielding the enthalpy change (∆*H*), the affinity (*K*_A_), and the stoichiometry of the β-CD-to-CEL complex (*n*). Table 2 compiles the calculated thermodynamic parameters of the CEL association with β-CD. The negative value of ∆*G* indicates that the complexation is thermodynamically favorable. The value of the entropy term is positive and larger than the enthalpy (|T∆*S*| > |∆*H*|); therefore, the process involved in the guest–CD interactions is entropy-driven, indicating hydrophobic interactions between CEL and β-CD [56]. As indicated by the value of the association constant, the interaction between the drug and the CD molecules is relatively strong (the stability of the complex is high). In addition, the stoichiometry of the complex formed between CEL and β-CD is close to the ratio of 1:2.

The preliminary ITC experiments allowed us to determine the optimal molar ratios for forming a complex between CEL and the appropriate CD. The complex was then prepared using the kneading method, taking 1:2 mol ration of the drug and β-CD. For this purpose, an appropriate amount of CD was wetted with water in a mortar and kneaded with a pestle to obtain a thick paste. A relevant amount of CEL was then dissolved in a small amount of acetone and mixed with CD in the mortar for twenty minutes. The mixture was dried to a constant weight, and the whole sample was powdered in the mortar. This method of preparing complexes is simple to perform, fast, and does not require expensive equipment. The effectiveness of the correct complex formation was confirmed during preliminary studies, and the developed methodology is consistent with the available literature data [16,17]. Thus, in the next stage of our research, the presence of the resulting CEL–β-CD inclusion complex was confirmed by DSC, FT-IR, ^1^H NMR, SEM and EDS techniques.

**Figure 3:**
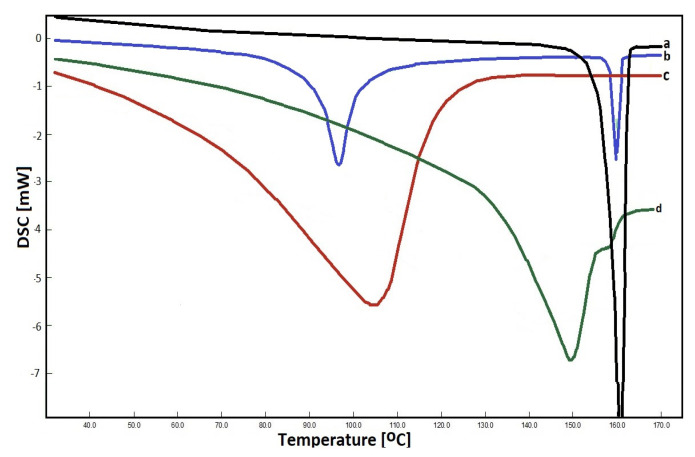
DSC thermograms registered for: CEL (a) physical mixture (b), β–CD (c) and CEL–β–CD complex (d).

DSC is a thermal analysis technique used to monitor various physical and chemical changes in a sample that are dependent on temperature [57]. It is used to study the melting and recrystallization of crystalline materials. Organic substances exhibit a certain melting range, the change of which can be correlated with impurities or other crystal ordering. In our experiments, the analysis of pure CEL, β-CD, and their complexes and physical mixture were performed by DSC, and registered thermograms are shown in Figure 3. The thermogram of CEL shows a sharp endothermic peak at 161 °C which corresponds to the decomposition temperature of crystalline CEL [58]. On the other hand, the β-CD thermogram shows a characteristic peak at 105 °C, caused by the evaporation of crystal water, which is embedded in the structure of CDs. In the case of the prepared complex, a new endothermic peak was obtained at about 150 °C, while in the case of the physical mixture prepared from CEL and β-CD, two peaks were present at 98 and 161 °C, which came from the individual components of the mixture.

Further tests (to prepare solid drug–CD binary systems) were also carried out. For this purpose, solutions of the drug in acetone and CD in water (in appropriate proportions) were stirred for 1 h and evaporated. In this case, in addition to the peaks corresponding to the pure components, an additional peak was obtained, around 150 °C. A number of tests of the kneading method were also performed using various solvents for wetting the drug, e.g., methanol, ethanol, acetonitrile. In each of them, DSC analysis showed drug residues not being part of the inclusion complex. Attempts were also made to create a solid mixture by grinding CEL and β-CD in appropriate proportions without first wetting and dissolving the ingredients, but no changes were observed in the thermogram.

**Figure 4:**
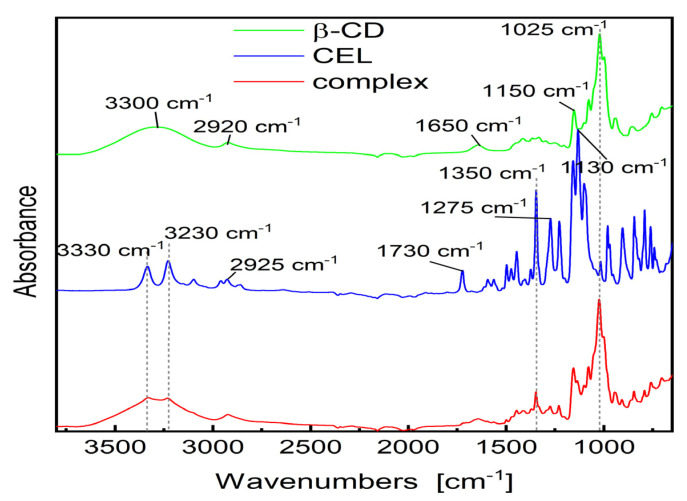
FT-IR spectra of the CEL, β-CD and analyzed CEL–β–CD complex.

Analysis of the FT-IR spectra of inclusion complexes helps determine host–guest interactions [59]. After encapsulation of the drug molecule by CD, changes in the spectra are observed, which result from the reduction in vibrations of free drug molecules, if its part is enclosed in the empty space of CD molecules [60,61]. The FT-IR spectra of pure CEL, β-CD and the obtained complex are shown in Figure 4. The following bands are observed on the β-CD spectra: wide band of stretching vibrations –O–H at about 3300 cm^−1^, aliphatic stretching band –C–H at 2920 cm^−1^, etc. The spectrum of CEL is characterized by the following bands: 3200–3300 cm^−1^ (–N–H stretching), about 2920 cm^−1^ (–C–H), 1730 cm^−1^ (–N–H deformation), 1500 cm^−1^ (C=C), 1275 cm^−1^ (–C–F), etc. In the region of approx. 3200–3350 cm^−1^ of the complex, overlapping of –O–H bands of CD and –N–H of the drug was observed. The bands at 1275 cm^−1^ (–C–F stretching vibration) and 1500 cm^−1^ (C=C stretching vibration) are visible, while the bands at 1730 cm^−1^ practically completely disappears, what indicates that this region of the drug molecule is located inside the CD cavity, and is shielded by the CD molecule. Comparing the FT-IR spectra of CEL, β-CD and CEL–β-CD, we may notice these changes, especially in the range of 1700–3400 cm^−1^. It may indicate the formation of hydrogen bonds between CEL and β-CD.

**Figure 5:**
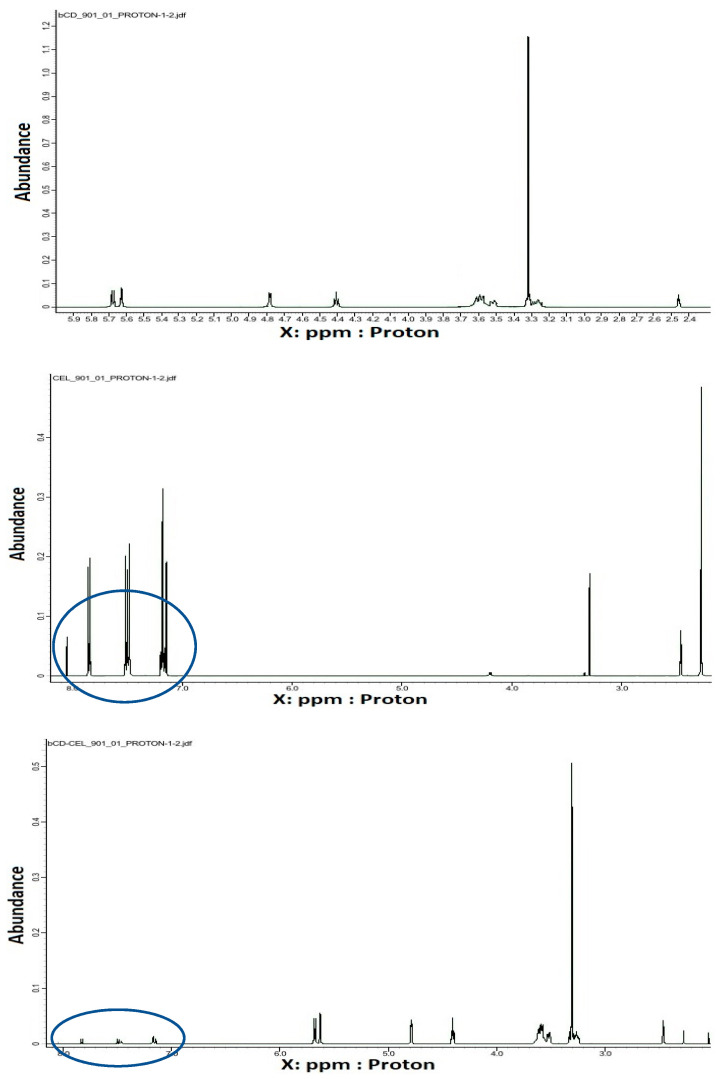
^1^H NMR spectra (399 MHz, 25 °C, DMSO-d_6_) registered for (**a**) β–CD, (**b**) CEL, and (**c**) CEL–β–CD.

**Figure 6:**
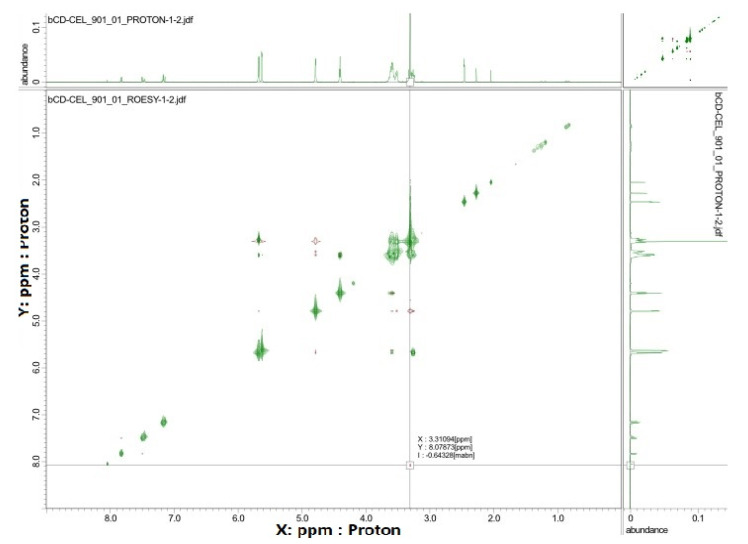
The ROESY spectrum (25 °C, DMSO-d_6_) registered for the CEL–β-CD.

NMR spectroscopy is a very useful tool in the study of the formation of inclusion complexes between CDs and various guest molecules. The drug molecule, which is enclosed in the hydrophobic cavity of the CD molecule, shows up in the spectrum as a shift of the drug and CD protons [62]. In general, the resonances of β-CD protons located in or near the cavity show shifts in the mixture. However, a slight shift can be observed for the resonance of protons outside the CD [63]. Figure 5 shows the ^1^H NMR spectra recorded for pure compounds and their inclusion complexes. Some differences in chemical shift values may indicate a change in the proton environment after the complex formation. The ^1^H NMR spectrum of CEL shows intense signals from protons at chemical shift σ between 7–8 ppm, and for β-CD in this range we notice no signals. However, in the case of the spectrum recorded for the CEL–β-CD complex, the proton signals in the chemical shift range 7–8 show very low intensity, which suggests that the process of drug inclusion by CD took place. The ROE correlation between the proton of the aryl moiety and the proton of the β-CD moiety can be observed using the ROESY spectrum, which provides a deeper insight into the stereochemical characteristic of inclusion complexes (Figure 6). It can be concluded that if the aryl moiety of the drug is included in the cavity of the CD, the ROE correlation between the protons of this moiety and the intracavitary protons of the β-CD moiety will be observed for host–guest size matching [63]. Then, it is possible to estimate the position of the aryl moiety in the β-CD cavity using assigned ROE correlations. The 2D-ROESY spectrum of CEL–β-CD complex given in Figure 6 shows the appearance of the ROE correlation signal, with coordinates: X 3.31094 ppm, Y 8.07873 ppm. The obtained results indicate that CEL penetrates into the β-CD cavity and an inclusion complex is formed.

**Figure 7:**
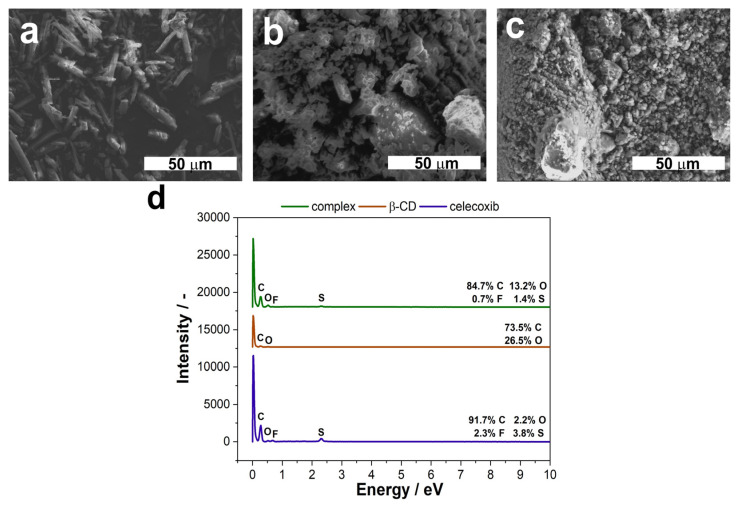
FE-SEM microphotographs of CEL (**a**), β–CD (**b**) and their complexes (**c**), along with their EDS spectra (**d**). The magnification on the SEM images is 1.0 k.

Finally, the SEM images of the CEL, β-CD and their complex powders were taken in order to determine their morphologies. Moreover, their elementary composition was assessed using EDS technique. The results are shown in Figure 7.

As can be seen in Figure 7, the tested molecules differ in their microstructure. CEL is in the form of needles (Figure 7a), whereas β-CD may be described as cauliflower-like crystals (Figure 7b). What is interesting is that the structure of the complex is more similar to that for the pure β-CD, though the crystals are smaller and more uniform (Figure 7c). This is consistent with the formulation composition, where the ratio between CEL and β-CD was 1:2. Furthermore, the effectiveness of the complexation process is proven by the EDS spectra (Figure 7d). The CEL molecule has sulfur and fluorine atoms in its structure, whereas β-CD has only carbon and oxygen. As can be seen from the spectra, we can see that peaks from both S and F are present, though the weight percentage is smaller than for the pure drug. Moreover, the percentage of oxygen increased, compared to the CEL.

Taking into account the results presented above, it can be stated that we were able to receive a CEL–β-CD inclusion complex by using a kneading method that was further used for the drug-release experiments.

### 3.2. Nanostructured TiO_2_ as a Carrier for CEL–β-CD Complexes

**Figure 8:**
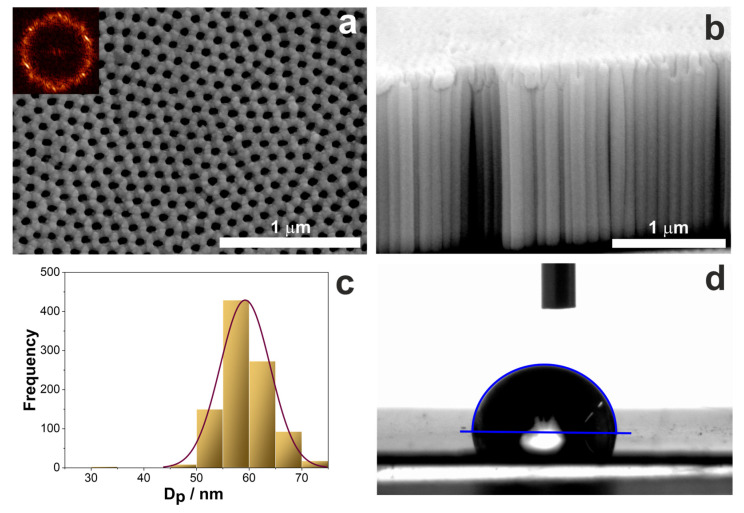
(**a**,**b**) FE-SEM microphotographs of the top (**a**) and cross-section (**b**) view of nanostructured TiO_2_ layer on Ti after anodization at 40 V in the ethylene glycol-based electrolyte. (**c**) Distribution of pore diameter and (**d**) exemplary image of the water droplet on the TiO_2_ surface during contact angle measurement.

Nanostructured titanium dioxide synthesized in the ethylene glycol-based solution with the addition of fluoride ions is characterized by the dual nanoporous/nanotubular structure [29]. The topography of such layers is strictly governed by the anodization conditions described in detail in our previous papers [29,64,65]. In this work, we used standard anodization parameters described in Section 2.6, which resulted in obtaining nanostructured layers characterized by a pore diameter (D_p_) of around 60 nm and a layer thickness of about 1.60 µm (Figure 8a,b). The pore arrangement is close to hexagonal, which is confirmed by the 2D fast Fourier transforms (FFTs) in the inset in Figure 8a, whereas the pore distribution is shown in the histogram (Figure 8c). The contact angle measurements showed that TiO_2_ layers are hydrophilic with a contact angle equal to 77° (Figure 8d). The results of the topography parameters and values of the contact angle and surface energy are compiled in Table 3.

Previously, we used such layers to deliver water-soluble and insoluble molecules, i.e., gentamicin and ibuprofen, respectively [51]. We have shown that the delivery of such molecules is possible and comprises two stages: firstly, the drug is released from the surface of the carrier (desorption part) and then from the inside of the pores (desorption and diffusion), which was described mathematically with the DDD model. The release profiles strictly depend on the solubility of the drug, which we have also shown previously. It is especially evident at the beginning of the release, where water-soluble molecules (e.g., gentamicin) release rapidly during the first minutes of the process (over 90% of the drug), followed by the continuous release of the rest of the gentamicin for a longer time. On the other hand, water-insoluble ibuprofen is released at a slower rate. Within the first hour, almost 80% of the drug is released, followed by a slower but constant release in the second stage.

**Figure 9:**
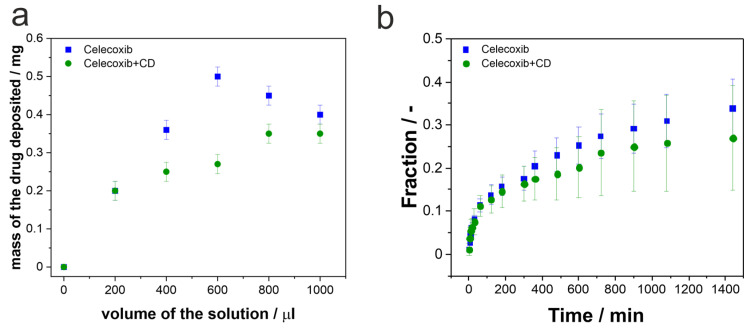
(**a**) The masses of the deposited drugs in correlation with the volume of the deposited solution. (**b**) CEL and CEL with β-CD release profiles showing changes in the fraction of the released drug in time. For each measurement, error bars represent the standard deviation for three independent samples.

In the present work, we wanted to discover how the nanostructured TiO_2_ layers will act as carriers for more complex systems, such as CEL and its inclusion complex with β-CD. We have applied the same procedure of loading the drug inside porous structures as previously, so the total amount of 1 mL of each solution was pipetted on the TiO_2_ carriers in portions of 200 µL [42,51]. After each cycle, the samples were dried in the air so that the solution would evaporate. Then, samples were cleaned with a paper towel soaked with ethanol so that the excess of the drug from the surface was removed, stored in the desiccator overnight, and weighed. Figure 9a shows the deposited molecule’s mass concerning the solution volume pipetted on the surface. It can be seen that for CEL, the pore saturation is achieved after 600 µL of the drug is deposited. Afterward, the mass of the drug drops, which may result from cleaning the surface with ethanol after the drying process. It may indicate that after the third deposition cycle, the excess of the drug is mainly present on the surface, and during the cleaning process, not only is the excess removed but also some of the drug from the inside of the pores. Slightly different behavior is observed for the inclusion complex. The mass of the deposited molecule increases gradually until it reaches a plateau, which may indicate that the capacity of the pores is saturated. What is more, applying the same cleaning process as for pure celecoxib does not change the loading process, since the complex is soluble in the water-enriched solution. Therefore, cleaning with ethanol will not dissolve the drug from the inside of the pores as much as for the CEL. Additionally, we have observed that due to the different solutions used for dissolving the tested molecules, the different wetting of the TiO_2_ surface was observed, and thus, different penetration of the pores. There was significantly less of the drug deposited on the surface of nanostructured TiO_2_ in the case of the complex compared to pure CEL. It may be summarized that by using the CEL complex, the loading process is facilitated due to its solubility.

The release process was carried out in the PBS with a pH of 7.4 at 37 °C for 24 h. The aliquots were collected at the pre-determined time and refilled with a fresh portion of PBS. Afterward, the drug concentrations were determined using the TLC method. The release profiles shown in Figure 9b depict the mean fraction of the drug released in time. The fraction was calculated according to Equation (3):

(3) $$F=\frac{m_{t}}{m_{0}}$$

where *F* is the fraction of the released drug; *m*_0_ is the mass of the drug loaded in the TiO_2_; and *m*_t_—a mass released at the time point.

As can be seen, the release profiles of CEL and the inclusion complex are similar. There is a fast release of the drugs from the surface at the beginning of the process, followed by a constant release from the pores for the rest of the time. Contrary to the previously shown data for ibuprofen and gentamicin [51], the initial release of CEL is 10%, during the first 30 min, despite the drug form. It is significantly lower than for the release of ibuprofen and gentamicin from the same carrier. In that case, after 30 min, almost 50% of ibuprofen and almost 100% of gentamicin were released in the same conditions. The differences between the pure drug and its complex start to show after 1.5 h of the release process. For the CEL–β-CD complex, the release is slightly slower than for pure CEL; however, the differences are not statistically significant. Nonetheless, after 24 h, only 25–30% of the drugs are released from the pores. Table 4 sums up the masses of the drugs at each stage of the process—the initially deposited amounts, the masses released during the burst stage of the process, and the amounts of the drugs released after 24 h.

The presented results confirm that nanostructured TiO_2_ may be used for the release of CEL and its complex with β-CD. The release profiles differ from those received for ibuprofen and gentamicin [51], showing a significant decrease in the released amounts of the drug during the whole process, which may be assigned to the size and structure of the molecules.

## 4. Conclusions

In the presented work, we have shown a simple and effective method to form an inclusion complex between CEL and β-CD, confirmed by the ITC, DSC, FT-IR, ^1^H NMR, SEM and EDS measurements. Moreover, we proved that such particles can be efficiently loaded inside TiO_2_ nanostructures as drug carriers. The release profiles indicated that both CEL and the CEL–β-CD complex are released in a dual mode: firstly, there is a burst release from the surfaces of the carrier (desorption), which is followed by the constant desorption and diffusion of the drugs from inside of the pores. For the presented set of drugs, there is no significant difference between the release profiles; however, the release of the complex is slightly slower than for pure CEL.

For that reason, there is still a lot to optimize in case of the proposed DDSs, such as the loading procedure, the capacity of TiO_2_, as well as the composition of the complexes. However, the presented preliminary studies showed that there is an alternative administration route for drugs like CEL that should be further explored in terms of, e.g., novel effective pain treatments after orthopedic surgeries. The presented results give the information that the inclusion complexes may be important in the development of various types of formulations. Not only do they improve solubility of the drugs, but also increase their bioavailability. Combined with the on-site delivery of the drugs, e.g., from the nanostructured carriers, it may open a wide range of possibilities, e.g., for their pharmacology and implantology.

## Figures and Tables

**Table 1:** The conditions applied for the anodization process of Ti supports.

Anodization Parameter	Value
Temperature	20 °C
Potential	40 V
Time of the 1st and 2nd anodization steps	2 h
Time of the 3rd anodization step	10 min

**Table 2:** Thermodynamic parameters of the interactions between β-CD and CEL determined by ITC experiments.

Sample	Temperature (°C)	N (Sites)	*K*_A_ (M^−1^)	∆*H* (kJ/mol)	∆*G* (kJ/mol)	T∆*S* (kJ/mol)	∆*S* (J/K mol)
β–CD	25.2	1.98	495.05	−5.69	−15.4	9.72	32.6

**Table 3:** Topography parameters, contact angle, and surface energy values for the TiO_2_@Ti nanostructures.

Pore Diameter/nm	Layer Thickness/µm	Water Contact Angle/°	Diiodomethane Contact Angle/°	Polar Component/mNm^−1^	Dispersive Component/mNm^−1^	Surface Energy/mNm^−1^
50 ± 3	1.60 ± 0.01	77.16 ± 8.47	41.07 ± 4.26	6.86	35.58	42.44

**Table 4:** The masses of the drugs loaded and released at the predetermined time points.

Time	Celecoxib mg	Celecoxib—β-Cyclodextrin mg
0	0.466 ± 0.112	0.350 ± 0.132
30 min	0.052 ± 0.028	0.027 ± 0.014
24 h	0.191 ± 0.076	0.094 ± 0.051

## Data Availability

Data sharing is not applicable.
